# Serum Trace Elements and Self-Reported Physician-Diagnosed Tumor History: A Secondary Cross-Sectional Analysis of the Exposure Study on Susceptible People (SPES) 

**DOI:** 10.3390/medicina62091663

**Published:** 2026-08-29

**Authors:** Carlo Buonerba, Paolo Montuori, Ernesto Esposito, Maria Triassi, Pellegrino Cerino

**Affiliations:** 1 Department of Public Health, University of Naples Federico II, Via Sergio Pansini 5, 80131 Naples, Italy; pmontuor@unina.it (P.M.); triassi@unina.it (M.T.); pellegrinocerino@gmail.com (P.C.); 2 Department of Health and Healthcare Services, Directorate-General of the Calabria Region, 88100 Catanzaro, Italy

**Keywords:** human biomonitoring, trace elements, serum metals, self-reported tumor history, chromium, Campania, cross-sectional study

## Abstract

*Background and Objectives*: SPES was a 2016–2017 regional biomonitoring program. We conducted a secondary cross-sectional analysis of associations between current serum trace-element distributions and heterogeneous, self-reported physician-diagnosed tumor history, rather than registry-confirmed or incident cancer. *Materials and Methods*: Among 4137 questionnaire responders, 86 reported a tumor history. Mixed-effects logistic models for 20 channels were adjusted for age, sex, smoking, and geographic cluster and pooled across 30 completed datasets. Benjamini–Hochberg FDR control was applied across 20 primary comparisons. *Results*: Higher Cr-52 was associated with lower prevalence odds of reported history (odds ratio per one-standard-deviation increase in log concentration, 0.751; 95% confidence interval, 0.601–0.937; *p* = 0.011; q = 0.226), but no channel met q < 0.05. Cr-52 was below the limit of quantification in 45/86 (52.3%) participants with reported history and 1646/4048 (40.7%) without. The inverse direction persisted in non-independent complementary analyses; none met the applicable FDR threshold. *Conclusions*: No channel met the FDR criterion. The Cr-52 pattern was nominal and exploratory. These prevalence associations with a heterogeneous, unvalidated self-reported outcome do not establish temporality, causality, chromium speciation, or a protective effect.

## 1. Introduction

Population exposure to metals arises through environmental, occupational, dietary, and lifestyle pathways. Several agents or compounds involving arsenic, beryllium, cadmium, hexavalent chromium, and nickel are carcinogenic to humans under defined chemical forms, routes, and exposure settings [1,2,3]. By contrast, total elemental concentration measured in serum is a matrix- and time-specific biomarker that does not identify chemical species, exposure source, historical dose, or the etiologic exposure window. Human biomonitoring can integrate multiple recent exposure pathways, but it does not by itself establish toxicity or causation [4]. European harmonization initiatives have further shown that biomarker concentrations vary with matrix, diet, smoking, occupation, and geography [5,6].

Prospective population evidence relating circulating trace elements to cancer remains sparse and inconsistent. In EPIC-Florence, prediagnostic serum chromium was not associated with breast cancer incidence in 150 cases and 150 matched controls, and more than half of chromium measurements were below the LOQ [7]. Other prospective cohorts have reported site- and matrix-specific associations and null findings for copper, zinc, arsenic, cadmium, and lead [8,9,10,11]. In contrast, the Dongfeng–Tongji study followed 4573 adults with type 2 diabetes, observed 541 medically confirmed incident cancers over a median 10.2 years, and reported an inverse association between baseline fasting-plasma chromium and cancer incidence [12]. These studies differ substantially in population, matrix, outcome, timing, and model specification.

Campania has long-standing concerns about waste-related and industrial exposures. Ecological studies have reported spatial variation in cancer mortality and incidence while emphasizing exposure misclassification, socioeconomic confounding, and the absence of individual biomarkers [13,14,15,16]. SPES was developed as an integrated regional program linking environmental characterization, population-based human biomonitoring, and biological effect assessment [17,18,19,20]. Its published geospatial analysis included 4188 participants and 20 serum analytical channels and documented pronounced small-area heterogeneity in current concentrations [21]; a companion analysis evaluated dietary correlates in the 4137 questionnaire responders [22]. What remained unknown was whether current total-serum trace-element distributions differed according to the uniformly collected tumor history item at the participant level. This question is distinct from established carcinogenic hazards of defined metal species and from prospective cancer risk estimation because the SPES endpoint is historical, heterogeneous, self-reported, and measured after or around diagnosis.

We hypothesized, without specifying a direction or causal effect, that current serum trace-element distributions might differ according to self-reported physician-diagnosed tumor history. We therefore conducted a post hoc secondary cross-sectional analysis of all 20 analytical channels, estimated prevalence associations with false discovery rate control, and examined a limited set of non-independent sensitivity analyses addressing covariate adjustment, left-censoring, and recent diagnosis. This study did not test incident cancer risk, historical etiologic exposure, or tumor development.

## 2. Materials and Methods

### 2.1. Study Design, Population, and Outcome Ascertainment

**Figure 1:**
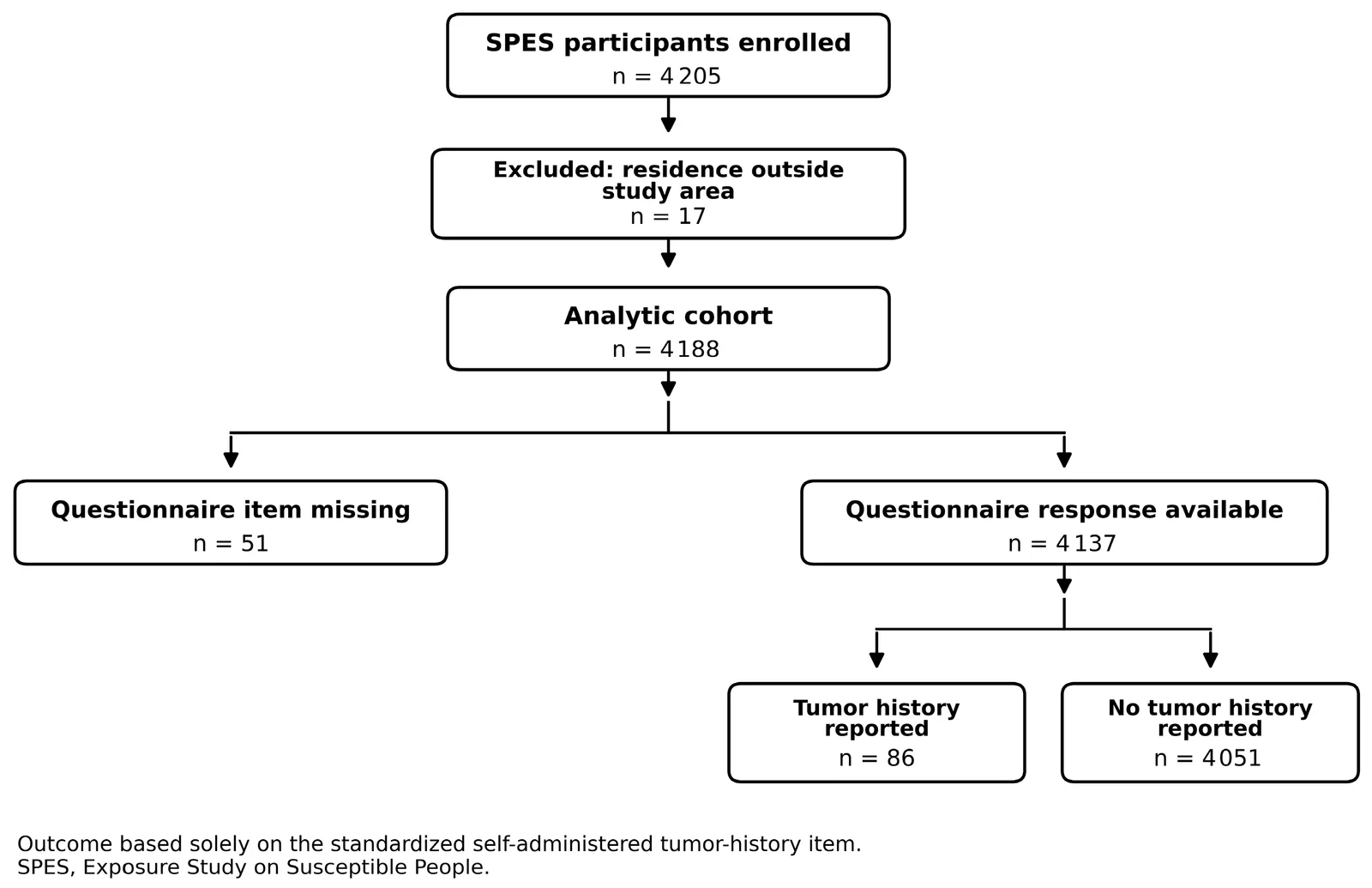
Participant flow for the self-reported physician-diagnosed tumor history analysis. The outcome was based solely on the standardized self-administered questionnaire item. SPES, Exposure Study on Susceptible People.

We conducted a post hoc secondary cross-sectional analysis of archived, de-identified SPES data from Campania, Italy. The parent program recruited adults from municipal registries across predefined geographic clusters in 2016–2017 and primarily targeted ages 20–49 years [18,19,21,22]. The analyzed dataset included a small number of enrolled participants outside that protocol range, with observed ages of 19–55 years. Recruitment was stratified by environmental pressure area, and the analytic cohort was not weighted to represent all adults in Campania. The original field protocol was not designed around the present tumor history association. Field, laboratory, and data processing procedures are described in peer-reviewed SPES publications [18,19,21,22]. The present analysis involved no new participant contact, registry linkage, specimen collection, or laboratory testing. The parent program enrolled 4205 participants; 17 residents outside the study area were excluded, leaving 4188 unique records. Of these, 4137 had an available tumor history response and 51 were excluded from outcome models because the response was missing. The sample size was fixed by the parent biomonitoring study; no outcome-specific or analyte-specific calculation was performed for this post hoc analysis. Reporting followed the Strengthening the Reporting of Observational Studies in Epidemiology (STROBE) statement, the STROBE Molecular Epidemiology extension (STROBE-ME), and the Statistical Analyses and Methods in the Published Literature (SAMPL) guidelines [23,24,25]. Participant flow is shown in Figure 1.

The outcome was derived from the standardized lifestyle questionnaire, which formed part of the broader EPIC-aligned SPES questionnaire framework [26]. The questionnaire recorded whether a physician had previously diagnosed a tumor and, when applicable, age at diagnosis and body site. Responses were classified as reported history, no reported history, or missing according to the stored questionnaire coding; the exact coding rules are provided in the Supplementary Materials. The outcome was not imputed. Because the item used the broad term “tumor”, it did not distinguish malignant, benign, or pre-invasive diagnoses and was not linked to registry or pathology records. We therefore defined the endpoint as self-reported history of a physician-diagnosed tumor; it should not be interpreted as confirmed cancer, malignant neoplasm history, or incident cancer. The source instrument did not collect separately verified benign and malignant outcomes, so retrospective reclassification would require unsupported assumptions. With only 86 reported histories, site-specific association models were not pursued. Validation studies suggest high specificity or positive predictive value for self-reported cancer, but only moderate and site-dependent sensitivity; reporting also varies with diagnostic definitions, sex, education, and time since diagnosis [27,28,29]. Body site and diagnosis age data were used only to describe the reported histories, not to define alternative outcomes or site-specific models.

### 2.2. Serum Trace-Element Measurements and Covariates

The 20 analytical channels represented 19 elements: lithium-7 (Li-7), beryllium-9, cadmium-111, arsenic-75, chromium-52 (Cr-52), cobalt-59, copper-63, iron-56, iron-57, mercury-202, manganese-55, molybdenum-98, nickel-60 (Ni-60), lead-208, antimony-121, selenium-78, strontium-88, thallium-205, vanadium-51, and zinc-66. Concentrations were reported in micrograms per liter except for iron-56, iron-57, and zinc-66, which were reported in milligrams per liter. The two iron isotope channels were retained for analytical reproducibility but were not interpreted as independent biological exposures. Serum was stored at −80 °C, thawed at room temperature, mixed by gentle rotation, and a 500 µL aliquot was diluted 1:10 (*v*/*v*) with a nitric acid diluent before analysis. A NexION 350 inductively coupled plasma mass spectrometry (ICP-MS) platform (PerkinElmer, Waltham, MA, USA), equipped with a concentric nebulizer (Meinhard Associates, Golden, CO, USA), a cyclonic spray chamber, a quartz torch, and a 2-mm internal-diameter quartz injector, was used; high-purity argon and helium were recorded as instrument gases. Element-specific five-point calibration curves were prepared daily, and yttrium-89, rhodium-103, and lutetium-175 were introduced online as internal standards. Calibration curves met R^2^ > 0.99. Each sample was analyzed in duplicate and the mean concentration was retained. Chemical blanks were monitored, LOQ estimation was based on the variability of 20 reagent blanks, and accuracy and precision were assessed using internal quality controls and certified reference materials [21,22]. The frozen extracts were used to record assay performance, below-LOQ status, analyte-specific LOQ, and the prespecified post-analytical high-value plausibility flag (HI20). Archived LOQs and assay support counts are reported in Supplementary Table S2B. Values below the LOQ were treated as censored observations rather than replaced by zero or a fixed fraction of the LOQ [30,31].

Tenfold dilution reduced the serum matrix burden, but dilution alone does not establish complete removal of potential carbon-derived interference at *m*/*z* 52. Exact collision/reaction cell settings, Cr-specific numerical recovery, and within-run or between-run precision summaries were unavailable in the archived materials used for this secondary analysis, precluding a new retrospective interference assessment.

Chromium speciation information was unavailable in the archived analytical dataset used for this secondary analysis. Cr-52 is therefore reported as a total serum analytical channel and cannot be assigned to Cr(III), Cr(VI), or another chromium species.

The core adjustment set was specified before fitting the channel-specific models using an a priori epidemiological confounding framework rather than data-driven significance testing. It included age, sex, cigarette smoking status, and geographic cluster because these factors may influence both serum trace-element distributions and the prevalence or reporting of tumor history; geographic cluster also reflects the SPES sampling structure and documented small-area heterogeneity. Age was modeled using a natural cubic spline with interior knots at 23, 35, and 47 years and boundary knots at the observed minimum and maximum ages of 19 and 55 years [32]. Sex was coded as female or male, smoking as never, former, or current, and the 21 SPES geographic clusters were represented by a random intercept. Body mass index, self-reported occupational exposure, and socioeconomic deprivation were included as plausible additional confounders in an extended-covariate sensitivity analysis. Potentially post-diagnostic factors—including treatment, current diet, inflammatory markers, organ function, and other clinical characteristics—were not included in the core model; where available, they could be consequences of the reported diagnosis rather than baseline confounders, and adjustment could introduce overcontrol or collider bias.

### 2.3. Statistical Analysis and Missing-Data Handling

Metal concentrations were analyzed in 30 completed datasets generated previously under the published SPES censoring-aware framework [21,33,34,35,36]. Quantified measurements were retained. Values below the LOQ and other eligible missing measurements had been completed using censored Gaussian, fixed Box–Cox, or interval-censored Gamma models selected for each channel. Unperformed Hg-202 and Ni-60 assays remained missing, so those analyses were restricted to participants with an assay result. For the other channels, limited assay nonperformance meeting the parent eligibility threshold was included in the parent procedure; model sample sizes can therefore be slightly larger than the corresponding raw assay counts. The present analysis was specified after the parent imputation run; however, the non-imputed tumor history item was already present as an auxiliary predictor and, for Cr-52, was retained after target-specific selection in both the censored Tobit and predictive-mean-matching components. The item itself was not imputed and remained unchanged across completed datasets. Predictor retention was target-specific rather than universal across channels. Outcome models were fitted separately in each completed dataset and combined using Rubin’s rules [33,36]. Additional analyses used observed quantification states and quantified concentrations only.

Participant characteristics were summarized by questionnaire response using the mean and standard deviation (SD), the median and interquartile range (IQR), or number and percentage, without hypothesis testing. We fitted a separate mixed-effects logistic regression for each analytical channel, with self-reported physician-diagnosed tumor history as the dependent variable and channel-specific standardized natural-log concentration, derived using constants common to all completed datasets and to the quantified-only analysis (Supplementary Methods), as the exposure. The models included age spline, sex, and smoking as fixed effects and geographic cluster as a random intercept. Mixed models were fitted with lme4 package (v2.0-6) [37]. Estimates are reported as prevalence odds ratios (ORs) per 1 SD increase in log concentration with channel-specific 95% confidence intervals (CIs). The Benjamini–Hochberg procedure was applied to the 20 primary *p* values; q < 0.05 indicated evidence after FDR control [38]. Convergence and singularity were assessed across the 30 completed datasets. Analyses were performed in R (v4.6.1; R Foundation for Statistical Computing, Vienna, Austria); complete package versions are reported in the Supplementary Materials.

Three complementary analyses addressed distinct sources of uncertainty. First, the extended-covariate analysis added body mass index, occupational exposure, and socioeconomic deprivation to the core model and was restricted to participants with valid BMI. Second, the quantification state analysis compared quantified results with values below the LOQ. This analysis does not estimate a concentration gradient; it is a decomposition of the primary exposure contrast that evaluates whether the observed pattern is expressed as a difference in the probability of being above the analytical quantification limit, rather than an independent replication. Third, the quantified-only analysis modeled continuous log concentration among measurements above the LOQ, thereby evaluating the direction of the association within the quantifiable range but in a selected and smaller participant subset. The two measured-data analyses were restricted to the 11 channels with at least 10% left-censoring and excluded unperformed assays and HI20 plausibility flags. Benjamini–Hochberg adjustment was applied separately to the 20 extended models and to the 11 eligible channels in each measured-data analysis. Assay completion for Hg-202 and Ni-60 was examined separately as an assay availability analysis.

### 2.4. Use of Generative Artificial Intelligence

OpenAI ChatGPT (GPT-5.6 Pro) and Anthropic Claude Opus 5 assisted with language editing, organization of manuscript materials, literature searching, and review of analysis code. They were not used to generate participant data, define the questionnaire outcome, select observations, or calculate statistical estimates. The authors verified the cited sources, code, numerical results, interpretations, and manuscript text and retain full responsibility for the work.

### 2.5. Ethics

The parent SPES study was conducted in accordance with the Declaration of Helsinki and approved by the Ethics Committee of IRCCS Fondazione G. Pascale (Commissioner Deliberation No. 590, 3 August 2016). All participants provided written informed consent. The present secondary analysis used de-identified data and involved no new participant contact, intervention, specimen collection, or laboratory testing.

## 3. Results

### 3.1. Study Population and Outcome Characteristics

The parent SPES program enrolled 4205 participants. After exclusion of 17 residents outside the study area, the frozen analytical dataset contained 4188 unique records. Of these, 4137 had an available tumor history response and entered the present outcome models: 86 (2.1%) reported that a physician had previously diagnosed a tumor and 4051 reported no such history. The remaining 51 participants had no usable response and were excluded because the outcome was not imputed (Figure 1; Supplementary Table S1). Supplementary Table S2B summarizes archived analyte-specific LOQs, assay support, HI20 flags, and primary model sample sizes. Participants reporting a tumor history were older (mean age, 38.2 versus 34.8 years) and more often female (72.1% versus 53.7%). Descriptive characteristics are shown in Table 1; no between-group hypothesis tests were performed.

The 86 reported histories covered a wide range of sites. The most common categories were breast (*n* = 20), thyroid/parathyroid (*n* = 17), female reproductive tract (*n* = 10), and central or peripheral nervous system or pituitary (*n* = 9). The median age at the reported diagnosis was 33.0 years [IQR, 25.0–39.8], and the median interval between diagnosis and SPES enrollment was 5.0 years [IQR, 2.0–8.0]. These data describe the questionnaire-defined outcome and were not used to derive site-specific models (Supplementary Tables S5 and S6).

### 3.2. Primary and Sensitivity Analyses

**Figure 2:**
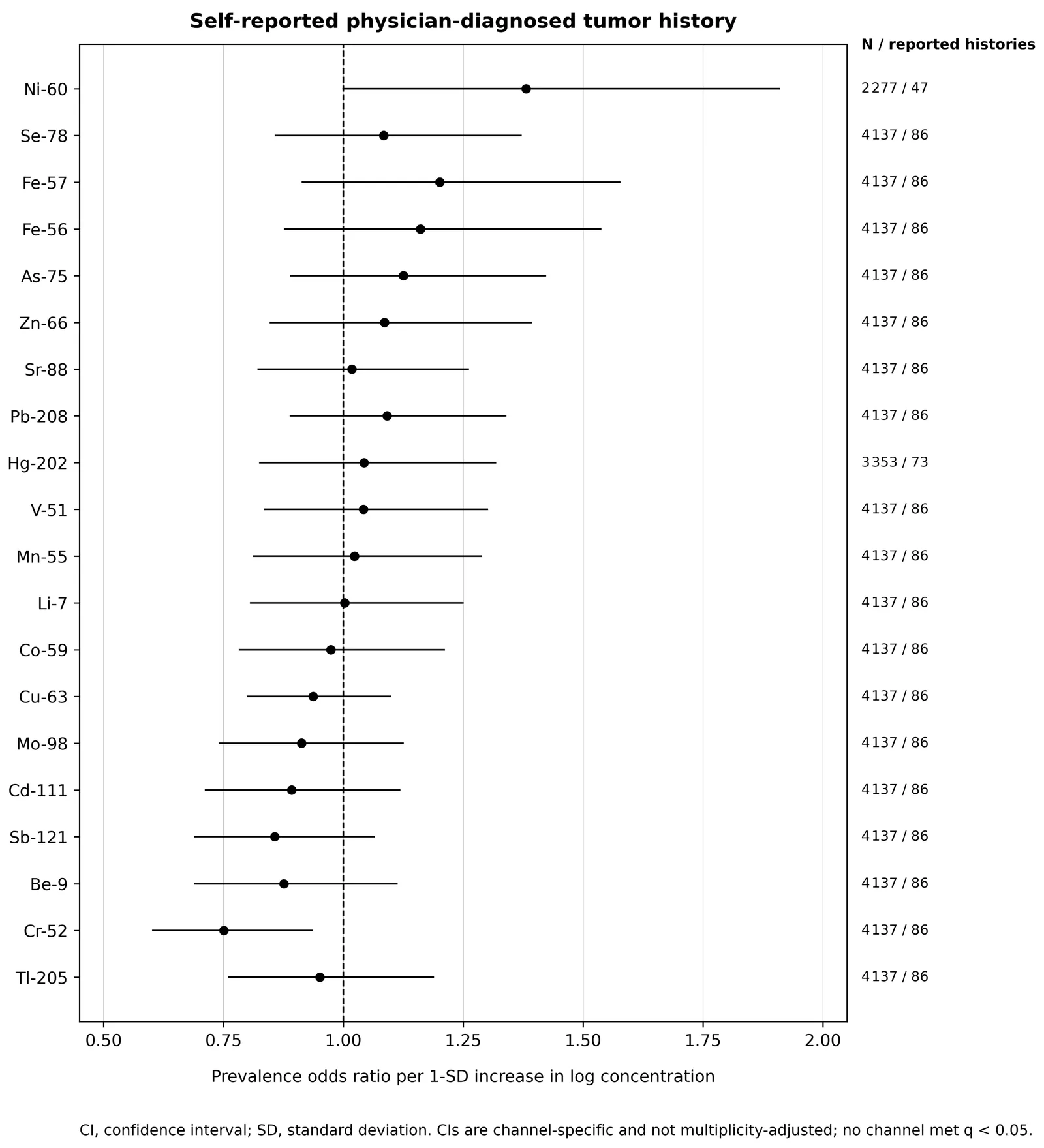
Adjusted prevalence odds ratios for self-reported physician-diagnosed tumor history per 1-standard-deviation (SD) increase in log-transformed serum trace-element concentration. Models were adjusted for age, sex, smoking, and geographic cluster. N/reported histories are shown for each analytical channel. The displayed 95% confidence intervals (CIs) are channel-specific and not multiplicity-adjusted; no channel met q < 0.05.

Cr-52 was the only nominal analyte-specific signal in the 20-channel primary analysis (OR per 1 SD increase in log concentration, 0.751; 95% CI, 0.601–0.937; *p* = 0.011), but it did not meet the FDR threshold (q = 0.226), and no channel met q < 0.05. Ni-60 was positive but imprecise among participants with an assay result (OR, 1.381; 95% CI, 0.998–1.911; *p* = 0.051; q = 0.513); confidence intervals for the remaining 18 channels included 1.0 (Figure 2; Supplementary Tables S2B and S3).

Cr-52 was below the LOQ in 45 of 86 participants with a reported history (52.3%) and in 1646 of 4048 participants without a reported history who had a performed assay (40.7%). Among quantified observations, median Cr-52 concentrations were 0.469 µg/L [IQR, 0.243–1.003] and 0.712 µg/L [IQR, 0.363–1.460], respectively; the corresponding geometric means were 0.452 and 0.685 µg/L. These quantified-value summaries are descriptive and do not replace the censoring-aware primary model (Supplementary Tables S2A and S4).

The Cr-52 estimate remained inverse after extended covariate adjustment among 4134 participants with valid BMI (OR, 0.754; 95% CI, 0.603–0.941; *p* = 0.013; q = 0.253). In the quantification state analysis, participants with a quantified Cr-52 result had lower odds of a reported tumor history than those with a result below the LOQ (OR, 0.616; 95% CI, 0.401–0.948). This finding shows that a substantial component of the primary pattern is expressed through quantification frequency and is not an independent replication of the continuous estimate. Among measurements above the LOQ, the quantified-only estimate was also inverse (OR, 0.375; 95% CI, 0.154–0.912), but it was based on 41 participants with a reported history and a selected subset of the exposure distribution. The primary and three prespecified sensitivity estimates each had 95% CIs excluding 1.0, but none of their corresponding q values were below 0.05. In the targeted time-related sensitivity analysis, exclusion of the 23 participants who reported a diagnosis within two years before enrollment left 4114 participants and 63 reported histories; the estimate remained inverse (OR, 0.684; 95% CI, 0.525–0.892; nominal *p* = 0.0049), and all 30 models converged without singularity or warnings. Taken together, the analyses identify a consistent difference in the current Cr-52 distribution—both in quantification status and among quantifiable values—without establishing temporality or a causal dose–response relationship. Table 2 summarizes the primary and prespecified sensitivity estimates.

Ni-60 did not show a stable pattern across analyses. The primary and extended-covariate estimates were positive but imprecise, whereas the quantification state analysis did not support an association. A larger estimate was observed among quantified concentrations only (OR, 2.698; 95% CI, 1.325–5.498; *p* = 0.006; q = 0.069), but this analysis included only 36 participants with a reported history and did not meet the FDR criterion. Assay completion was similar in the two questionnaire groups, reducing concern that availability differed substantially by outcome; however, the geographic random-intercept variance was at or near zero in all 30 completed datasets. In targeted logistic models with cluster represented by fixed categorical effects, Hg-202 remained null (OR, 1.003; 95% CI, 0.764–1.317; nominal *p* = 0.982), whereas the Ni-60 estimate was attenuated and more imprecise (OR, 1.314; 95% CI, 0.933–1.851; nominal *p* = 0.118). Ni-60 therefore remains a secondary exploratory and specification-sensitive observation (Supplementary Section S2.2 and Tables S7–S11). All 30 Cr-52 primary models converged without singular fits.

## 4. Discussion

Across the full 20-channel family, no association met the prespecified FDR criterion. A single nominal association in this setting remains compatible with chance. The primary contribution is therefore not evidence that chromium protects against tumors, but complete FDR-controlled reporting of a predominantly null panel in a population-based biomonitoring resource. Cr-52 was the only nominal analyte-specific pattern, and its inverse direction was observed in non-independent complementary analyses of the completed distribution, quantification state, and quantified values. This internal consistency supports hypothesis generation but does not provide independent validation or override q = 0.226.

Directional context is provided by Dongfeng–Tongji, but the two studies are not symmetric. That cohort included 4573 older adults with type 2 diabetes who were cancer-free at baseline, measured 12 fasting-plasma metals before diagnosis, documented 541 incident cancers over a median 10.2 years, and estimated chromium in a Cox model mutually adjusted for the other 11 metals (HR per 1 SD higher log chromium, 0.88; 95% CI, 0.82–0.94) [12]. The SPES included a younger population-based sample, measured current total Cr-52 in serum after or around the reported diagnosis, used a broad unvalidated self-reported prevalent outcome, and fitted separate channel-specific logistic models. The shared direction is therefore directional concordance only, not replication or evidence that the coefficients estimate the same quantity.

Reverse causation is a central limitation. Serum was collected after or around the questionnaire-reported diagnosis, exact diagnosis and collection dates were unavailable, and an interval of zero completed years does not establish within-year ordering. Excluding the 23 diagnoses reported within two years of enrollment did not attenuate the Cr-52 estimate, but this limited robustness analysis cannot establish that the biomarker preceded disease. Longer-term treatment, dietary or occupational change, altered body composition, inflammation, renal handling, metabolic change, survivor selection, outcome misclassification, residual confounding, and chance remain plausible explanations.

Chemical interpretation is also limited. Experimental and supplementation studies have examined insulin signaling, oxidative stress, and inflammatory markers [39,40,41,42,43,44,45], but they do not establish cancer prevention. EFSA concluded that an essential metabolic function of Cr(III) in humans and beneficial effects of chromium intake in healthy people had not been substantiated [46]. Circulating chromium may reflect recent intake, absorption, protein binding, renal handling, insulin-related physiology, inflammation, treatment, and other metabolic features [47,48]. In randomized trials, chromium picolinate did not improve insulin sensitivity relative to placebo in healthy adults, and chromium treatment produced no glycemic benefit in poorly controlled, insulin-treated type 2 diabetes [49,50]; within the chromium-treated group in Masharani et al., higher achieved serum chromium was associated with worsening insulin sensitivity [49]. Human serum speciation work identified chromium–transferrin, a trivalent complex, as the main detectable chromium species at physiological concentrations, while experimentally added Cr(VI) was reduced slowly and remained partly detectable after 48 h [51]. However, chromium speciation information was unavailable in the archived SPES analytical dataset, so this literature cannot determine which species, or what proportions of species, contributed to the total-serum Cr-52 signal. The SPES result therefore supports neither a protective Cr(III) effect, chromium deficiency, lower Cr(VI) exposure, nor chromium supplementation.

Prospective findings outside Dongfeng–Tongji are not uniform. EPIC-Florence found no association between prediagnostic serum chromium and breast cancer incidence in 150 cases and 150 matched controls, with more than half of chromium measurements below the LOQ [7]. A later exploratory EPIC-Florence analysis of 50 cases each of lung, bladder, and prostate cancer with matched controls likewise found no significant case–control differences in the measured serum metal panel [52]. Moreover, the additional copper, lead, zinc, manganese, and nickel signals reported in Dongfeng–Tongji were not reproduced as primary SPES patterns. Population, matrix, cancer-site composition, metabolic status, timing, censoring, and multimetal adjustment may all contribute to cross-study differences.

Cr-52 was below the LOQ in 52.3% of participants with a reported history and 40.7% of those reporting no history with an assay performed. Accordingly, the primary result should not be interpreted as a simple continuous dose–response relationship: part of the pattern reflects a difference in the probability of quantification. The quantified-only estimate shows that the direction was not created solely by completed sub-LOQ values, but it is conditional on being above the LOQ and included only 41 reported histories. The most defensible summary is a current total-serum Cr-52 distributional difference, not an independently replicated concentration gradient. Ni-60 was less stable: only 47 participants with a reported history had an assay, the primary and extended confidence intervals included 1.0, the random-effect variance was at the boundary, and the larger quantified-only estimate was not supported by the other analyses.

This study has several additional limitations. The cross-sectional design cannot establish temporality or causality. The outcome was heterogeneous, self-reported, and not validated through pathology or registry linkage; the broad term “tumor” did not distinguish malignant, benign, or pre-invasive diagnoses, and only 86 histories were available, limiting precision and precluding site-specific inference. An approximate precision-calibrated calculation indicated that the primary design had 80% power only for ORs of about 0.73 or 1.37 at α = 0.05, so modest associations could have been missed. Twenty primary comparisons required FDR control, and the panel-wide null results should not be interpreted as proof of absence. Residual confounding and post-diagnostic change remain possible.

Analytical and missing-data limitations also require caution. Several channels were substantially left-censored; quantified-only analyses apply to selected subsets; and Hg-202 and Ni-60 were restricted to measured participants. Total-serum channels do not identify species, source, historical dose, or the relevant etiologic window. The parent imputation used target-specific predictor selection: tumor history was retained in both Cr-52 components but not in every channel-specific model. Where it was not retained, imputed cells—not observed quantified values—were not directly conditioned on the outcome. Measured-data sensitivities and low censoring elsewhere reduce, but do not eliminate, this concern. Original instrument files, complete batch records, exact collision/reaction cell settings, interference-correction equations, and Cr-specific recovery and precision summaries were unavailable, precluding retrospective verification of isotope-specific analytical uncertainty.

Strengths include population-based registry sampling within the SPES, a uniformly administered questionnaire item, reporting of all 20 channels rather than selective presentation, a parsimonious primary adjustment set, published censoring-aware data processing, explicit multiplicity control, observed-data sensitivity analyses, and detailed assay support and model diagnostics. The present analysis also adds a participant-level health history layer to the published SPES geospatial and food-source findings [21,22]. These strengths improve transparency but do not overcome the outcome, timing, and analytical limitations.

An appropriate next study would use prospectively collected or archived prediagnostic specimens, validated registry-linked and site-specific cancer outcomes, species-appropriate biomarker matrices, adequate event numbers, contemporary interference control documentation, and prespecified multimetal, confounding, and multiplicity plans. Such a design could determine whether the current SPES pattern precedes diagnosis, marks a metabolic or nutritional phenotype, or reflects changes after diagnosis. Until then, the result should remain a transparent, predominantly null, hypothesis-generating data point rather than evidence of a causal or protective chromium effect.

## 5. Conclusions

In this post hoc secondary cross-sectional analysis, no serum analytical channel met the prespecified FDR criterion. Cr-52 was the only nominal exploratory pattern, and its inverse direction across non-independent complementary analyses does not establish temporality, causality, chromium species, deficiency, or a protective effect. The principal contribution is complete reporting of a population-based 20-channel panel and definition of a hypothesis for prospective testing. Future studies should use prediagnostic specimens, validated registry-linked outcomes, species-appropriate matrices, adequate event numbers, and prespecified multimetal and multiplicity control.

## Figures and Tables

**Table 1:** Participant characteristics according to self-reported physician-diagnosed tumor history.

Characteristic	Overall (*n* = 4137)	Tumor History Reported (*n* = 86)	No Tumor History Reported (*n* = 4051)
Participants, *n*	4137	86	4051
Age, years	34.9 (8.8)	38.2 (7.8)	34.8 (8.8)
Female sex	2236 (54.0%)	62 (72.1%)	2174 (53.7%)
Smoking: never	2099 (50.7%)	40 (46.5%)	2059 (50.8%)
Smoking: former	762 (18.4%)	23 (26.7%)	739 (18.2%)
Smoking: current	1276 (30.8%)	23 (26.7%)	1253 (30.9%)
Body mass index, kg/m^2^	25.4 (4.5); 24.6 [22.3–27.7]	25.4 (4.3); 24.7 [22.4–28.1]	25.4 (4.5); 24.6 [22.3–27.7]
Obesity (BMI ≥ 30 kg/m^2^)	577 (14.0%)	12 (14.0%)	565 (14.0%)
Occupational exposure: yes	822 (19.9%)	15 (17.4%)	807 (19.9%)
Socioeconomic deprivation index	33.3 [8.3–37.5]	33.3 [18.8–58.3]	29.2 [8.3–37.5]
High-impact area	2393 (57.8%)	40 (46.5%)	2353 (58.1%)
Medium-impact area	1147 (27.7%)	31 (36.0%)	1116 (27.5%)
Low-impact area	597 (14.4%)	15 (17.4%)	582 (14.4%)

Age is shown as mean (SD); body mass index as mean (SD) and median [IQR]; socioeconomic deprivation as median [IQR]; categorical variables as *n* (%). Abbreviations: BMI, body mass index; IQR, interquartile range; SD, standard deviation. Percentages use non-missing denominator. Body mass index was available for 4134 participants (86 with and 4048 without reported history); all other displayed variables were available for all 4137 questionnaire responders. No hypothesis tests were performed.

**Table 2:** Cr-52 estimates across the primary and sensitivity analyses.

Analysis	N/Reported Histories	OR (95% CI)	*p* Value	q Value
Primary	4137/86	0.751 (0.601–0.937)	0.011	0.226
Extended-covariate sensitivity analysis	4134/86	0.754 (0.603–0.941)	0.013	0.253
Quantification state sensitivity analysis	4134/86	0.616 (0.401–0.948)	0.028	0.303
Quantified-only sensitivity analysis	2443/41	0.375 (0.154–0.912)	0.030	0.168

Primary and extended-covariate q values were calculated across 20 channels; quantification state and quantified-only q values were calculated across the 11 high-censoring channels eligible for those analyses. Below the limit of quantification (LOQ) was the reference category in the quantification state analysis. Abbreviations: CI, confidence interval; OR, odds ratio; SD, standard deviation. Benjamini–Hochberg q values were calculated from unrounded *p* values; recomputation from the displayed three-decimal *p* values may differ slightly.

## Data Availability

The individual-level data are not publicly available because they contain health and biomonitoring information subject to ethics, privacy, and data governance restrictions. De-identified data supporting the findings may be made available by the SPES data custodian upon reasonable request, approval of the competent governance bodies, and execution of a data use agreement. Final analysis scripts and public reproducibility materials—including the input schema, data dictionary, software environment, aggregate expected outputs, numerical checks, synthetic smoke-test materials, and SHA-256 checksums—are publicly available as release v1.0.0 of the SPES analysis-code package in Zenodo at https://doi.org/10.5281/zenodo.22071985 (accessed on 1 August 2026). Individual-level data and the archived multiple-imputation object remain restricted under SPES data governance requirements.

## Supplementary Materials

The following supporting information can be downloaded at https://www.mdpi.com/article/10.3390/medicina62091663/s1. Supplementary Materials: Serum Trace Elements and Self-Reported Physician-Diagnosed Tumor History: A Secondary Cross-Sectional Analysis of the Population-Based SPES Biomonitoring Study; Table S1. Questionnaire outcome counts; Table S2A. Assay completion and quantification according to questionnaire response; Table S2B. Archived analyte-specific limits of quantification, assay support, censoring, HI20 plausibility flags, and primary model sample sizes among 4137 questionnaire responders; Table S3. Complete primary channel-specific associations; Table S4. Descriptive summaries among quantified observations; Table S5. Self-reported tumor site categories among 86 reported histories; Table S6. Age at diagnosis and interval from diagnosis to SPES enrollment; Table S7. Primary mixed-model diagnostics across 30 completed datasets; Table S8. Hg-202 and Ni-60 assay completion according to questionnaire response; Table S9. Complete extended-covariate sensitivity analysis; Table S10. Complete quantification state sensitivity analysis; Table S11. Complete quantified-only sensitivity analysis.
