# Supplementary material for: Serum Trace Elements and Self-Reported Physician-Diagnosed Tumor History: A Secondary Cross-Sectional Analysis of the Exposure Study on Susceptible People (SPES)
Source: Medicina (Kaunas). 2026 Aug 29;62(9):1663. doi: 10.3390/medicina62091663 (PMC13609303; doi:10.3390/medicina62091663)
Supplement: Supplementary file 1 [file medicina-62-01663-s001.zip › medicina-4510894-supplementary.pdf]

Serum Trace Elements and Self-Reported Physician-Diagnosed Tumor History: A Secondary Cross-Sectional Analysis of the Population-Based SPES Biomonitoring Study

Supplementary Materials

Carlo Buonerba[1,*], Paolo Montuori[1], Ernesto Esposito[2], Maria Triassi[1] and Pellegrino Cerino[1]

[1] Department of Public Health, University of Naples Federico II, Via Sergio Pansini 5, 80131 Naples, Italy

[2] Department of Health and Healthcare Services, Directorate-General of the Calabria Region, 88100 Catanzaro, Italy

[*] Correspondence: Carlo Buonerba; Email: carbuone@hotmail.com

Reference numbers correspond to the reference list in the main manuscript. Abbreviations: BMI, body mass index; CI, confidence interval; FDR, false discovery rate; HI20, post-analytical high-value plausibility flag; ICP-MS, inductively coupled plasma mass spectrometry; IQR, interquartile range; LOD, limit of detection; LOQ, limit of quantification; OR, odds ratio; QC, quality control; SD, standard deviation; SPES, Exposure Study on Susceptible People.

S1. Supplementary Methods

S1.1. Cohort, Outcome, and Descriptive Site Harmonization

The Exposure Study on Susceptible People (SPES) field campaign was conducted in 2016–2017 as an integrated regional biomonitoring program. The present work is a post hoc secondary analysis of its archived, de-identified dataset. The analytic cohort comprised 4,188 participants after exclusion of 17 enrollees residing outside the study area. The parent protocol primarily targeted adults aged 20–49 years, whereas observed ages in the analysis dataset ranged from 19 to 55 years. The present analysis included the 4,137 participants with an available response to the standardized self-administered tumor history item. A total of 86 reported that a physician had previously diagnosed a tumor, 4,051 reported no such history, and 51 participants with a blank response were excluded from outcome models. When a tumor history was reported, the stored field recorded age at diagnosis; a positive age value denoted a reported history, zero or an explicit negative response denoted no history, and a blank field was treated as missing. The outcome was not imputed. Because the source instrument collected one broad tumor history item rather than separately verified malignant and benign diagnoses, retrospective reclassification would require unsupported assumptions. The item did not distinguish malignant, benign, or pre-invasive diagnoses and was not linked to registry or pathology information. The estimate is therefore the prevalence OR for a reported physician-diagnosed tumor history, not cancer incidence or confirmed malignant neoplasm history. With only 86 reported histories, site-specific association models were not pursued. Years since diagnosis were calculated as age at SPES enrollment minus reported age at diagnosis, both recorded in completed years. Exact diagnosis and blood collection dates were unavailable; therefore, a derived interval of zero years indicates occurrence within the same completed age-year and does not establish exact within-year ordering. All derived intervals were non-negative. Free-text body site responses were harmonized for descriptive purposes using a deterministic, rule-based keyword crosswalk developed for this secondary analysis and applied uniformly to all participants with a reported history. The crosswalk was frozen before final descriptive tabulation. This harmonization did not establish histology or malignant behavior, alter outcome status, or support site-specific association models. The participant-level raw-to-category mapping was retained in restricted audit materials.

S1.2. Laboratory Measurements and Missing-Data Handling

Twenty serum analytical channels representing nineteen elements were evaluated: Li-7, Be-9, Cd-111, As-75, Cr-52, Co-59, Cu-63, Fe-56, Fe-57, Hg-202, Mn-55, Mo-98, Ni-60, Pb-208, Sb-121, Se-78, Sr-88, Tl-205, V-51, and Zn-66. Fe-56 and Fe-57 were retained as separate inductively coupled plasma mass spectrometry (ICP-MS) channels for analytical reproducibility but were not interpreted as independent biological exposures. Concentrations were expressed in µg/L except for Fe-56, Fe-57, and Zn-66, which were expressed in mg/L. Serum was stored at −80 °C, thawed at room temperature, mixed by gentle rotation, and a 500 µL aliquot was diluted 1:10 (v/v) with a nitric acid diluent before analysis. Analyses used a NexION 350 ICP-MS platform (PerkinElmer) equipped with a concentric nebulizer, cyclonic spray chamber, quartz torch, and 2 mm internal-diameter quartz injector. High-purity argon and helium were recorded as instrument gases. Element-specific five-point calibration curves were prepared daily; yttrium-89, rhodium-103, and lutetium-175 were introduced online as internal standards. Calibration curves had $R^2 > 0.99$, each sample was analyzed in duplicate, and the mean was retained. Chemical blanks were monitored, LOQ estimation was based on the variability of 20 reagent blanks, and accuracy and precision were assessed using internal quality controls and certified reference materials [21–22].

The frozen analytical extract preserved, for each channel, the concentration, assay-performed indicator, below-LOQ indicator, analyte-specific LOQ, and prespecified post-analytical high-value plausibility flag (HI20). HI20 was not an instrument or batch quality control failure category. Flagged source values were set to missing and were not retained as observed concentrations or used for model fitting or donor selection; eligible cells were completed under the archived missing-data workflow. Quantified measurements were retained, and values below the LOQ were treated as left-censored observations rather than replaced by zero or a fixed fraction of the LOQ. Archived LOQs were constant within channels and are reported with assay support counts in Table S2B. Unperformed Hg-202 and Ni-60 assays were not imputed, so those channels were analyzed on measured subsets. For the other channels, limited assay nonperformance meeting the parent eligibility threshold could be included under the published SPES missing-data framework [21–22].

Tenfold dilution reduced the serum matrix burden, but dilution alone does not establish complete removal of potential carbon-derived interference at m/z 52. Exact collision/reaction cell settings, Cr-specific numerical recovery, and within-run or between-run precision summaries were unavailable in the archived materials used for this secondary analysis, precluding a new retrospective interference assessment.

Chromium speciation information was unavailable in the archived analytical dataset used for this secondary analysis. Cr-52 is therefore reported as a total-serum analytical channel and cannot be assigned to Cr(III), Cr(VI), or another chromium species. Laboratory measurements were interpreted within the original SPES analytical framework, and small isotope channel differences require caution.

We used 30 completed datasets previously generated with the published censoring-aware SPES framework [21,33–36]. The parent procedure used censored Gaussian, fixed Box–Cox, or interval-censored Gamma models according to the prespecified family of each analytical channel. The present secondary analysis was specified after that imputation run; however, the standardized questionnaire item used to define tumor history was already present in the parent dataset as a non-imputed auxiliary variable and remained unchanged across completed datasets. Archived target-specific predictor selection records confirmed that the tumor history indicator was retained in both the censored Tobit component for below-LOQ Cr-52 observations and the predictive-mean-matching component for eligible non-censored Cr-52 missing values. Retention was target-specific rather than universal across all channel-specific models. Where the item was not retained, the imputed cells—not the observed quantified concentrations—were generated without direct conditioning on tumor history status, creating potential imputation analysis uncongeniality that could attenuate or otherwise bias an association under some missing-data structures. Outcome models were fitted separately in each completed dataset, and estimates were combined using Rubin's rules. The quantification state and quantified-only analyses used observed assay states or measurements above the LOQ and therefore did not depend on completed sub-LOQ concentrations. Unperformed Hg-202 and Ni-60 assays were not imputed.

S1.3. Statistical Models, Sensitivity Analyses, Diagnostics, and Software

For each analytical channel, we fitted the following mixed-effects logistic model, with covariates and random effects defined below:

$$\mathrm{logit}\{\Pr(Y_i = 1)\} = \beta_0 + \beta_1 Z_i + f(\mathrm{age}_i) + \beta\mathrm{sex}\ \mathrm{sex}_i + \beta\mathrm{smoking}\ \mathrm{smoking}_i + \mathrm{bcluster}[i]$$

Y_i denotes a reported physician-diagnosed tumor history, and Z_i is the channel-specific standardized natural-log concentration expressed per one standard-deviation increase. For each channel, the mean and standard deviation of the natural-log concentration were calculated separately in each completed dataset. The 30 means were averaged to obtain one channel-specific log-mean constant, and the 30 standard deviations were averaged to obtain one channel-specific log-standard-deviation constant. The same pair of constants was applied to every completed dataset and to the quantified-only analysis. The function f(age) is a natural cubic spline with interior knots at 23, 35, and 47 years and boundary knots at 19 and 55 years. The term bcluster[i] is a random intercept for the 21 SPES geographic clusters. The 20 primary p values formed one inferential family, and Benjamini–Hochberg q values were calculated across those 20 tests.

The extended-covariate analysis added body mass index, self-reported occupational exposure, and socioeconomic deprivation to the primary adjustment set; q values were calculated across 20 channels. BMI was available for 4,134 participants. One BMI was already missing in the submitted analysis; during the revision audit, two additional outcome-negative values derived from implausible adult height entries were set to missing. The quantification state analysis compared quantified measurements with values below the LOQ among the 11 channels with at least 10% left-censoring. It evaluates whether the primary pattern is expressed as a difference in the probability of being above the quantification limit and should be interpreted as a decomposition of the exposure contrast rather than an independent replication. The quantified-only analysis used the same 11 channels but modeled continuous log concentration only among quantified measurements; it evaluates the direction within the quantifiable range but applies to a selected, smaller subset. The two measured-data analyses excluded unperformed assays and HI20 plausibility flags and used separate 11-channel FDR adjustments. Assay completion for Hg-202 and Ni-60 was evaluated separately to assess whether assay availability differed by outcome. A targeted Cr-52 analysis excluded participants whose reported diagnosis occurred 0–2 years before enrollment. For Hg-202 and Ni-60, logistic models were fitted without a random effect, both without a cluster term and with cluster represented by fixed categorical effects. The assay completion models and these targeted analyses were summarized using nominal p values and did not define additional discovery families. Benjamini–Hochberg q values were calculated from unrounded p values; recomputation from displayed three-decimal p values may differ slightly.

To calibrate statistical precision, approximate minimum detectable odds ratios were calculated from the pooled standard error of the exactly reproduced primary Cr-52 coefficient. For a two-sided Wald test, the absolute detectable log odds ratio was calculated as the sum of the two-sided critical value and the target-power critical value, multiplied by the pooled standard error; exponentiation yielded the decrease and increase bounds. Calculations were reported for 80% power at $\alpha = 0.05$ and at $\alpha = 0.0025$ as a deliberately conservative 20-comparison benchmark. The latter was not treated as a fixed Benjamini–Hochberg threshold, and the calculation was neither an a priori power analysis nor retrospective observed power.

For each analyte, convergence, singularity, warnings, and geographic random-intercept variance were summarized across the 30 completed datasets. A singular fit indicates that the random-intercept variance was estimated at or near zero; the exposure coefficient can still be estimated. Pooling included every finite coefficient–variance pair. Five of six-hundred primary fits produced optimizer-gradient warnings but yielded finite, non-singular estimates: one each for Cu-63, Tl-205, and V-51, and two for Zn-66. In an audit recalculation, excluding these five fits changed the corresponding pooled ORs by less than 0.03% and did not alter any interpretation. In the extended-covariate family after the BMI validity recheck, 37 of 600 fits produced scaled-gradient warnings. Targeted refitting with independent optimizers yielded materially concordant coefficients, standard errors, and pooled estimates; the maximum OR change across channels was 0.0012%, no FDR threshold classification changed, and the rounded Cr-52 result was unchanged. The expected boundary singular fits remained confined to Hg-202 and Ni-60. Analyses were conducted in R 4.6.1 using readxl 1.5.0, mice 3.19.0, lme4 2.0-6, Matrix 1.7-5, splines, and supporting packages. Exact software and run information are retained in the reproducibility archive.

S2. Supplementary Results

S2.1. Outcome, Assay Support, and Primary Results

Cr-52 was the only nominal analyte-specific signal in the primary panel: its 95% confidence interval (CI) excluded 1.0 (odds ratio (OR), 0.751; 95% CI, 0.601–0.937; $p = 0.011$; $q = 0.226$). The primary Ni-60 estimate was imprecise (OR, 1.381; 95% CI, 0.998–1.911; $p = 0.051$; $q = 0.513$). No primary channel met $q < 0.05$. Tables S1, S2A, S2B, S3, and S4 report outcome counts, assay support, all primary estimates, and descriptive summaries among measurements above the LOQ; Table S2B consolidates archived LOQs, assay support, HI20 flags, and primary model sample sizes for all 20 channels.

Table S1. Questionnaire outcome counts.

Response	N	Analytic handling
Tumor history reported	86	Included as outcome-positive
No tumor history reported	4,051	Included as outcome-negative
Missing	51	Excluded; outcome was not imputed
Total	4,188	SPES analytic cohort

Tumor history reported denotes a positive self-administered questionnaire response; no tumor history reported denotes an explicit negative response.

Table S2A. Assay completion and quantification according to questionnaire response.

Analyte	History reported: assay n	History reported: <LOQ, n (%)	History reported: quantified, n (%)	No history: assay n	No history: <LOQ, n (%)	No history: quantified, n (%)
Li-7	86	0 (0.0%)	86 (100.0%)	4,051	24 (0.6%)	4,024 (99.3%)
Be-9	86	66 (76.7%)	20 (23.3%)	4,051	2,807 (69.3%)	1,244 (30.7%)
Cd-111	86	40 (46.5%)	46 (53.5%)	4,051	1,810 (44.7%)	2,241 (55.3%)
As-75	86	4 (4.7%)	82 (95.3%)	4,050	291 (7.2%)	3,759 (92.8%)
Cr-52	86	45 (52.3%)	41 (47.7%)	4,048	1,646 (40.7%)	2,402 (59.3%)
Co-59	86	1 (1.2%)	85 (98.8%)	4,048	32 (0.8%)	4,016 (99.2%)
Cu-63	86	0 (0.0%)	86 (100.0%)	4,048	1 (0.0%)	4,047 (100.0%)
Fe-56	86	0 (0.0%)	86 (100.0%)	4,048	8 (0.2%)	4,040 (99.8%)
Fe-57	86	0 (0.0%)	86 (100.0%)	4,048	7 (0.2%)	4,041 (99.8%)
Hg-202	73	21 (28.8%)	52 (71.2%)	3,280	845 (25.8%)	2,430 (74.1%)
Mn-55	86	7 (8.1%)	79 (91.9%)	4,050	407 (10.0%)	3,643 (90.0%)
Mo-98	86	16 (18.6%)	70 (81.4%)	4,051	601 (14.8%)	3,447 (85.1%)
Ni-60	47	11 (23.4%)	36 (76.6%)	2,230	676 (30.3%)	1,548 (69.4%)
Pb-208	86	58 (67.4%)	28 (32.6%)	4,051	2,876 (71.0%)	1,171 (28.9%)
Sb-121	86	37 (43.0%)	49 (57.0%)	4,051	1,469 (36.3%)	2,577 (63.6%)
Se-78	86	0 (0.0%)	86 (100.0%)	4,051	1 (0.0%)	4,050 (100.0%)
Sr-88	86	0 (0.0%)	86 (100.0%)	4,051	1 (0.0%)	4,050 (100.0%)
Tl-205	86	22 (25.6%)	64 (74.4%)	4,051	1,041 (25.7%)	3,009 (74.3%)
V-51	86	11 (12.8%)	75 (87.2%)	4,048	458 (11.3%)	3,581 (88.5%)
Zn-66	86	0 (0.0%)	86 (100.0%)	3,871	1 (0.0%)	3,870 (100.0%)

Group totals were 86 participants with a reported history and 4,051 participants reporting no such history. Percentages are calculated among participants with an assay performed for that channel. For some channels, assay-performed N exceeds below-LOQ plus quantified N because a small number of source values carried an HI20 plausibility flag or were otherwise unusable. HI20 was a post-analytical plausibility flag, not an instrument or batch quality control failure. For channels other than Hg-202 and Ni-60, limited assay nonperformance meeting the parent eligibility threshold could be included under the published SPES missing-data framework; therefore, the primary model N can be slightly larger than the raw assay-performed N. Table S2B reports archived LOQs, overall assay support, HI20 flag counts, and primary model N for all channels. LOQ, limit of quantification.

Table S2B. Archived analyte-specific limits of quantification, assay support, censoring, HI20 plausibility flags, and primary model sample sizes among 4,137 questionnaire responders.

Analyte	Unit	Archived LOQ	Assay performed, n	Assay unperformed, n (%)	Below LOQ among performed, n (%)	Quantified, n	HI20 flags, n	Primary model N
Li-7	µg/L	0.0048	4,137	0 (0.0%)	24 (0.6%)	4,110	3	4,137
Be-9	µg/L	0.0015	4,137	0 (0.0%)	2,873 (69.4%)	1,264	0	4,137
Cd-111	µg/L	0.0010	4,137	0 (0.0%)	1,850 (44.7%)	2,287	0	4,137
As-75	µg/L	0.0015	4,136	1 (0.02%)	295 (7.1%)	3,841	0	4,137
Cr-52	µg/L	0.0015	4,134	3 (0.07%)	1,691 (40.9%)	2,443	0	4,137
Co-59	µg/L	0.0016	4,134	3 (0.07%)	33 (0.8%)	4,101	0	4,137
Cu-63	µg/L	0.3007	4,134	3 (0.07%)	1 (0.02%)	4,133	0	4,137
Fe-56	mg/L	0.0007	4,134	3 (0.07%)	8 (0.2%)	4,126	0	4,137
Fe-57	mg/L	0.0006	4,134	3 (0.07%)	7 (0.2%)	4,127	0	4,137
Hg-202	µg/L	0.1192	3,353	784 (19.0%)	866 (25.8%)	2,482	5	3,353
Mn-55	µg/L	0.0096	4,136	1 (0.02%)	414 (10.0%)	3,722	0	4,137
Mo-98	µg/L	0.0099	4,137	0 (0.0%)	617 (14.9%)	3,517	3	4,137
Ni-60	µg/L	0.0212	2,277	1,860 (45.0%)	687 (30.2%)	1,584	6	2,277
Pb-208	µg/L	0.0182	4,137	0 (0.0%)	2,934 (70.9%)	1,199	4	4,137
Sb-121	µg/L	0.0020	4,137	0 (0.0%)	1,506 (36.4%)	2,626	5	4,137
Se-78	µg/L	0.1114	4,137	0 (0.0%)	1 (0.02%)	4,136	0	4,137
Sr-88	µg/L	0.0193	4,137	0 (0.0%)	1 (0.02%)	4,136	0	4,137
Tl-205	µg/L	0.0012	4,137	0 (0.0%)	1,063 (25.7%)	3,073	1	4,137
V-51	µg/L	0.0087	4,134	3 (0.07%)	469 (11.3%)	3,656	9	4,137
Zn-66	mg/L	0.0004	3,957	180 (4.4%)	1 (0.03%)	3,956	0	4,137

Archived LOQs were the constant analyte-specific thresholds stored in the frozen SPES analytical extract and used in the archived workflow; they were not newly derived or independently revalidated for this revision.Non-assay-performed percentages use all 4,137 questionnaire responders as the denominator; percentages below the LOQ use performed assays as the denominator. Quantified counts exclude below-LOQ results and HI20 source values. HI20 was a post-analytical high-value plausibility flag, not an instrument or batch quality control failure. HI20 source values were set to missing and were not retained as observed concentrations or used for model fitting or donor selection; eligible cells were completed under the archived missing-data workflow. Limited assay nonperformance was eligible for completion only when it did not exceed the parent 5% responder threshold; unperformed Hg-202 and Ni-60 assays were not imputed, so their primary models remained restricted to measured responders. This table summarizes archived LOQ and assay support metadata and is not intended to replace analyte-specific recovery or precision data. Separate instrument-level LODs and numerical analyte-specific recovery and precision summaries were unavailable in the secondary analysis archive.

Table S3. Complete primary channel-specific associations.

Analyte	N	Reported histories, n	Adjusted OR	95% CI	p value	q value
Li-7	4,137	86	1.003	0.805–1.251	0.977	0.977
Be-9	4,137	86	0.876	0.689–1.113	0.279	0.777

Analyte	N	Reported histories, n	Adjusted OR	95% CI	p value	q value
Cd-111	4,137	86	0.892	0.711–1.119	0.324	0.777
As-75	4,137	86	1.125	0.889–1.423	0.327	0.777
Cr-52	4,137	86	0.751	0.601–0.937	0.011	0.226
Co-59	4,137	86	0.974	0.782–1.212	0.812	0.918
Cu-63	4,137	86	0.937	0.799–1.100	0.428	0.777
Fe-56	4,137	86	1.161	0.876–1.538	0.299	0.777
Fe-57	4,137	86	1.201	0.913–1.578	0.190	0.777
Hg-202	3,353	73	1.043	0.824–1.319	0.726	0.907
Mn-55	4,137	86	1.023	0.811–1.289	0.849	0.918
Mo-98	4,137	86	0.913	0.741–1.126	0.395	0.777
Ni-60	2,277	47	1.381	0.998–1.911	0.051	0.513
Pb-208	4,137	86	1.091	0.888–1.340	0.407	0.777
Sb-121	4,137	86	0.857	0.689–1.066	0.166	0.777
Se-78	4,137	86	1.084	0.857–1.372	0.502	0.797
Sr-88	4,137	86	1.018	0.821–1.262	0.872	0.918
Tl-205	4,137	86	0.951	0.760–1.189	0.659	0.907
V-51	4,137	86	1.042	0.834–1.302	0.718	0.907
Zn-66	4,137	86	1.086	0.846–1.393	0.518	0.797

Adjusted prevalence odds ratios (ORs) are reported per one channel-specific standard-deviation (SD) higher natural-log concentration. Models included age, sex, smoking, and a geographic cluster random intercept. q values are Benjamini–Hochberg adjustments across the 20 primary models. All estimates were combined across 30 completed datasets. CI, confidence interval. Benjamini–Hochberg q values were calculated from unrounded p values; recomputation from displayed three-decimal p values may differ slightly.

Table S4. Descriptive summaries among quantified observations.

Analyte	Unit	History reported: quantified, n	History reported: median [IQR]	History reported: geometric mean	No history: quantified, n	No history: median [IQR]	No history: geometric mean
Li-7	µg/L	86	1.017 [0.713–1.322]	1.005	4,024	1.027 [0.739–1.495]	1.060
Be-9	µg/L	20	0.0575 [0.0348–0.126]	0.0524	1,244	0.0527 [0.0189–0.0962]	0.0412
Cd-111	µg/L	46	0.0353 [0.0173–0.122]	0.0394	2,241	0.0507 [0.0192–0.141]	0.0496
As-75	µg/L	82	1.503 [0.653–4.103]	1.682	3,759	1.701 [0.756–3.619]	1.648
Cr-52	µg/L	41	0.469 [0.243–1.003]	0.452	2,402	0.712 [0.363–1.460]	0.685
Co-59	µg/L	85	0.968 [0.729–1.215]	0.925	4,016	0.944 [0.715–1.218]	0.904
Cu-63	µg/L	86	980.6 [846.1–1,218.8]	988.4	4,047	989.3 [841.6–1,164.0]	987.4
Fe-56	mg/L	86	1.246 [0.939–1.524]	1.160	4,040	1.214 [0.905–1.582]	1.155
Fe-57	mg/L	86	1.249 [0.902–1.547]	1.177	4,041	1.204 [0.893–1.578]	1.152
Hg-202	µg/L	52	1.995 [0.965–3.806]	1.942	2,430	1.643 [0.765–3.210]	1.544
Mn-55	µg/L	79	1.145 [0.761–1.579]	1.027	3,643	1.203 [0.786–1.683]	1.094

Analyte	Unit	History reported: quantified, n	History reported: median [IQR]	History reported: geometric mean	No history: quantified, n	No history: median [IQR]	No history: geometric mean
Mo-98	µg/L	70	0.914 [0.545–1.298]	0.875	3,447	0.910 [0.573–1.285]	0.825
Ni-60	µg/L	36	3.463 [1.858–5.819]	2.924	1,548	1.918 [0.935–3.576]	1.793
Pb-208	µg/L	28	0.387 [0.136–0.821]	0.335	1,171	0.299 [0.110–0.663]	0.284
Sb-121	µg/L	49	0.162 [0.118–0.245]	0.152	2,577	0.166 [0.109–0.237]	0.156
Se-78	µg/L	86	191.8 [168.2–225.0]	192.0	4,050	196.2 [161.5–232.2]	190.1
Sr-88	µg/L	86	28.38 [22.65–36.97]	29.04	4,050	28.31 [22.65–35.55]	28.67
Tl-205	µg/L	64	0.0268 [0.0175–0.0541]	0.0285	3,009	0.0316 [0.0177–0.0579]	0.0319
V-51	µg/L	75	0.488 [0.323–0.748]	0.504	3,581	0.475 [0.295–0.748]	0.440
Zn-66	mg/L	86	1.400 [1.199–1.616]	1.395	3,870	1.430 [1.215–1.659]	1.385

Summaries are restricted to measurements above the LOQ and do not represent the full distribution when left-censoring is substantial. No unadjusted hypothesis tests were performed. IQR, interquartile range; LOQ, limit of quantification.

S2.2. Outcome Characteristics and Model Diagnostics

The 86 reported histories covered diverse sites and diagnosis times. The most common categories were breast, thyroid/parathyroid, female reproductive tract, and central or peripheral nervous system or pituitary. All 30 Cr-52 primary models converged and none were singular. For Hg-202 and Ni-60, the geographic random-intercept variance was estimated at or near zero in all 30 primary models; this boundary estimate indicates no detectable residual between-cluster variance and does not prevent estimation of the fixed exposure coefficient. In logistic models without a cluster term, the pooled estimates were essentially identical to the boundary mixed models: Hg-202, N = 3,353 with 73 reported histories, OR 1.043 (95% CI, 0.824–1.319; nominal $p = 0.726$); and Ni-60, N = 2,277 with 47 reported histories, OR 1.381 (95% CI, 0.998–1.911; nominal $p = 0.051$). With cluster represented by fixed categorical effects, Hg-202 remained null (OR 1.003; 95% CI, 0.764–1.317; nominal $p = 0.982$), whereas Ni-60 was attenuated and more imprecise (OR 1.314; 95% CI, 0.933–1.851; nominal $p = 0.118$). All 30 fits in both alternative specifications converged without warnings. Because the Ni-60 fixed-cluster model estimated many geographic parameters with only 47 reported histories, it was treated as a robustness check rather than a replacement primary model. Crude assay completion was similar between questionnaire groups for Ni-60 and only modestly different for Hg-202 (Tables S5–S8).

Table S5. Self-reported tumor site categories among 86 reported histories.

Self-reported site category	N	Percentage of 86 reported histories
Breast	20	23.3%
Thyroid/parathyroid	17	19.8%
Female reproductive tract	10	11.6%
Central/peripheral nervous system and pituitary	9	10.5%
Head/neck and other soft-tissue sites	7	8.1%
Skin/melanocytic	7	8.1%
Lung/mediastinum	4	4.7%
Gastrointestinal/hepatopancreatic	3	3.5%
Testis	3	3.5%
Hematologic/bone marrow	2	2.3%
Kidney/adrenal	2	2.3%
Unspecified	1	1.2%
Multiple sites (breast and uterus)	1	1.2%

Body site text was assigned to broad descriptive categories using the deterministic, rule-based crosswalk described in Section S1.1. The categories do not establish histology or malignant behavior and were not used in the association models.

Table S6. Age at diagnosis and interval from diagnosis to SPES enrollment.

Measure	Value
Age at diagnosis, median [IQR], years	33.0 [25.0–39.8]
Age at diagnosis, range, years	1–47
Years since diagnosis, median [IQR]	5.0 [2.0–8.0]
Diagnosis 0–2 years before enrollment	23
Diagnosis >2–5 years before enrollment	24
Diagnosis >5–10 years before enrollment	25
Diagnosis >10 years before enrollment	14

Table S7. Primary mixed-model diagnostics across 30 completed datasets.

Analyte	Models fitted, n/30	Converged, n/30	Singular fits, n/30	Models with warnings, n/30	Random-intercept variance, median	Minimum	Maximum
As-75	30/30	30/30	0/30	0/30	0.0358	0.0356	0.0360
Be-9	30/30	30/30	0/30	0/30	0.0393	0.0374	0.0425
Cd-111	30/30	30/30	0/30	0/30	0.0436	0.0386	0.0468
Co-59	30/30	30/30	0/30	0/30	0.0337	0.0336	0.0338
Cr-52	30/30	30/30	0/30	0/30	0.0390	0.0361	0.0410
Cu-63	30/30	29/30	0/30	1/30	0.0358	0.0350	0.0360
Fe-56	30/30	30/30	0/30	0/30	0.0350	0.0349	0.0351
Fe-57	30/30	30/30	0/30	0/30	0.0344	0.0344	0.0345
Hg-202	30/30	30/30	30/30	0/30	<0.0001	<0.0001	<0.0001
Li-7	30/30	30/30	0/30	0/30	0.0337	0.0336	0.0337
Mn-55	30/30	30/30	0/30	0/30	0.0325	0.0312	0.0347
Mo-98	30/30	30/30	0/30	0/30	0.0376	0.0363	0.0402
Ni-60	30/30	30/30	30/30	0/30	<0.0001	<0.0001	<0.0001
Pb-208	30/30	30/30	0/30	0/30	0.0308	0.0291	0.0322
Sb-121	30/30	30/30	0/30	0/30	0.0509	0.0448	0.0567
Se-78	30/30	30/30	0/30	0/30	0.0324	0.0324	0.0324
Sr-88	30/30	30/30	0/30	0/30	0.0325	0.0325	0.0325
Tl-205	30/30	29/30	0/30	1/30	0.0321	0.0312	0.0331
V-51	30/30	29/30	0/30	1/30	0.0350	0.0347	0.0352
Zn-66	30/30	28/30	0/30	2/30	0.0313	0.0308	0.0318

Cluster variance is the variance of the geographic cluster random intercept. Values below 0.0001 are displayed as <0.0001. For Hg-202 and Ni-60, singularity reflected a random-intercept variance estimated at or near zero; the fixed exposure coefficient remained estimable. Pooling included all finite coefficient–variance pairs. Five warning-bearing fits across Cu-63, Tl-205, V-51, and Zn-66 were retained because their coefficients and variances were finite and the fits were non-singular; excluding them in an audit recalculation changed the corresponding pooled ORs by less than 0.03%.

Table S8. Hg-202 and Ni-60 assay completion according to questionnaire response.

Analyte	History reported: assay n/N (%)	No history: assay n/N (%)	Adjusted OR	95% CI
Hg-202	73/86 (84.9%)	3,280/4,051 (81.0%)	1.217	0.657–2.254
Ni-60	47/86 (54.7%)	2,230/4,051 (55.0%)	0.796	0.489–1.297

Adjusted odds ratios (ORs) compare the odds of assay completion in participants with a reported history versus those reporting no such history using the same age, sex, smoking, and geographic cluster structure as the assay availability model. CI, confidence interval.

S2.3. Sensitivity Analyses

The Cr-52 estimate remained inverse after extended covariate adjustment among 4,134 participants with valid BMI (OR, 0.754; 95% CI, 0.603–0.941; $p = 0.013$; $q = 0.253$). In the quantification state analysis, a quantified result was associated with lower odds of a reported tumor history than a result below the LOQ (OR, 0.616; 95% CI, 0.401–0.948), showing that an important component of the primary contrast is expressed through quantification frequency. In the quantified-only analysis, the estimate was also inverse (OR, 0.375; 95% CI, 0.154–0.912), but only 41 participants with a reported history had a quantifiable Cr-52 value. None of the corresponding q values were below 0.05. The results therefore support a consistent current Cr-52 distributional difference, not an independently replicated or causal concentration–response relationship. For Ni-60, only the quantified-only estimate excluded 1.0. That estimate was based on 36 participants with a reported history, was not supported by the primary, extended-covariate, or quantification state analyses, and did not meet the FDR threshold (Tables S9–S11).

Conditional on the observed primary Cr-52 pooled standard error of 0.11321, the approximate 80% power detectable bounds were OR ≤ 0.728 or ≥ 1.373 at two-sided $\alpha = 0.05$ and OR ≤ 0.646 or ≥ 1.549 using $\alpha = 0.0025$ as a conservative 20-comparison benchmark. After excluding the 23 participants who reported a diagnosis 0–2 years before enrollment, the analysis included 4,114 participants and 63 reported histories and remained inverse (OR 0.684; 95% CI, 0.525–0.892; nominal $p = 0.0049$); all 30 models converged, none were singular, and no warnings occurred. This targeted analysis did not define an additional FDR family, does not constitute independent replication, and does not establish temporality.

Table S9. Complete extended-covariate sensitivity analysis.

Analyte	N	Reported histories, n	Adjusted OR	95% CI	p value	q value
Li-7	4,134	86	1.014	0.811–1.267	0.904	0.904
Be-9	4,134	86	0.877	0.690–1.115	0.285	0.792
Cd-111	4,134	86	0.896	0.715–1.124	0.344	0.792
As-75	4,134	86	1.139	0.900–1.443	0.279	0.792
Cr-52	4,134	86	0.754	0.603–0.941	0.013	0.253
Co-59	4,134	86	0.972	0.779–1.213	0.799	0.882
Cu-63	4,134	86	0.928	0.790–1.091	0.366	0.792
Fe-56	4,134	86	1.165	0.877–1.546	0.292	0.792
Fe-57	4,134	86	1.205	0.915–1.586	0.185	0.792
Hg-202	3,352	73	1.062	0.838–1.345	0.621	0.882
Mn-55	4,134	86	1.024	0.813–1.291	0.838	0.882
Mo-98	4,134	86	0.916	0.743–1.128	0.408	0.792
Ni-60	2,277	47	1.367	0.985–1.898	0.062	0.618
Pb-208	4,134	86	1.085	0.883–1.334	0.435	0.792
Sb-121	4,134	86	0.862	0.693–1.074	0.186	0.792
Se-78	4,134	86	1.076	0.850–1.361	0.543	0.835
Sr-88	4,134	86	1.026	0.825–1.277	0.815	0.882
Tl-205	4,134	86	0.959	0.767–1.199	0.714	0.882

Analyte	N	Reported histories, n	Adjusted OR	95% CI	p value	q value
V-51	4,134	86	1.038	0.830–1.298	0.742	0.882
Zn-66	4,134	86	1.084	0.843–1.394	0.529	0.835

The extended-covariate analysis added body mass index, occupational exposure, and socioeconomic deprivation to the primary adjustment set. The analysis used the 4,134 participants with valid BMI for the 18 generally completed channels; Hg-202 and Ni-60 remained restricted to their measured subsets. q values are Benjamini–Hochberg adjustments across 20 channels. All estimates were combined across 30 completed datasets. A targeted alternative-optimizer audit yielded materially concordant pooled estimates and no change in FDR classification. Abbreviations: CI, confidence interval; OR, odds ratio; SD, standard deviation.

Table S10. Complete quantification state sensitivity analysis.

Analyte	N	Reported histories, n	Adjusted OR	95% CI	p value	q value
Be-9	4,137	86	0.675	0.406–1.120	0.128	0.487
Cd-111	4,137	86	0.881	0.572–1.358	0.566	0.778
Cr-52	4,134	86	0.616	0.401–0.948	0.028	0.303
Hg-202	3,348	73	0.865	0.517–1.449	0.583	0.778
Mn-55	4,136	86	1.198	0.547–2.625	0.651	0.778
Mo-98	4,134	86	0.726	0.416–1.265	0.258	0.710
Ni-60	2,271	47	1.349	0.680–2.677	0.392	0.778
Pb-208	4,133	86	1.164	0.735–1.843	0.518	0.778
Sb-121	4,132	86	0.713	0.459–1.108	0.133	0.487
Tl-205	4,136	86	0.998	0.610–1.634	0.994	0.994
V-51	4,125	86	0.883	0.463–1.687	0.707	0.778

Below the limit of quantification (LOQ) was the reference category. Analyses included only performed assays without an HI20 plausibility flag. q values are Benjamini–Hochberg adjustments across the 11 eligible high-censoring channels. Abbreviations: CI, confidence interval; OR, odds ratio. Benjamini–Hochberg q values were calculated from unrounded p values; recomputation from displayed three-decimal p values may differ slightly.

Table S11. Complete quantified-only sensitivity analysis.

Analyte	N	Reported histories, n	Adjusted OR	95% CI	p value	q value
Be-9	1,264	20	1.476	0.682–3.192	0.323	0.507
Cd-111	2,287	46	0.673	0.371–1.221	0.193	0.424
Cr-52	2,443	41	0.375	0.154–0.912	0.030	0.168
Hg-202	2,482	52	1.331	0.875–2.026	0.182	0.424
Mn-55	3,722	79	0.771	0.413–1.441	0.416	0.572
Mo-98	3,517	70	1.160	0.626–2.150	0.637	0.752
Ni-60	1,584	36	2.698	1.325–5.498	0.006	0.069
Pb-208	1,199	28	1.100	0.695–1.743	0.684	0.752
Sb-121	2,626	49	0.914	0.375–2.227	0.842	0.842
Tl-205	3,073	64	0.780	0.478–1.272	0.319	0.507
V-51	3,656	75	1.389	0.869–2.221	0.170	0.424

Odds ratios (ORs) are reported per the primary channel-specific 1-standard-deviation (SD) increment and are restricted to quantified measurements. q values are Benjamini–Hochberg adjustments across the 11 eligible high-censoring channels. CI, confidence interval. Benjamini–Hochberg q values were calculated from unrounded p values; recomputation from displayed three-decimal p values may differ slightly.
